# A polymorphism at *IGF1* locus is associated with anemia

**DOI:** 10.18632/oncotarget.16132

**Published:** 2017-03-11

**Authors:** Maria Adelaide Marini, Gaia Chiara Mannino, Teresa Vanessa Fiorentino, Francesco Andreozzi, Francesco Perticone, Giorgio Sesti

**Affiliations:** ^1^ Department of Systems Medicine, University of Rome Tor Vergata, Rome, Italy; ^2^ Department of Medical and Surgical Sciences, University Magna-Græcia of Catanzaro, Catanzaro, Italy

**Keywords:** insulin-like growth factor 1, hemoglobin, rs35767, anemia, single nucleotide polymorphism, Pathology Section

## Abstract

*In vitro* and *in vivo* studies suggest that IGF-1 has a role in erythropoiesis. There is evidence that the rs35767 C/T polymorphism near *IGF1* is associated with plasma IGF-1 levels. We investigated the effect of this polymorphism on hemoglobin (Hb) concentration and anemia. The study group comprised 3286 adult Whites. The rs35767 polymorphism was screened using a TaqMan allelic discrimination assay. The rs35767 polymorphism was not associated with age, gender, BMI, waist circumference, smoking, blood pressure, plasma glucose, HbA1c, type 2 diabetes, HOMA-IR, hsCRP, eGFR, and lipid profile. Erythrocyte sedimentation rate (ESR), fibrinogen, and fasting insulin levels were significantly lower in TT genotype carriers compared with C allele carriers. Hb concentration was significantly higher in carriers of the TT genotype compared with C allele carriers, and a lower proportion of TT carriers had anemia. As compared with TT genotype carriers, those bearing the CC genotype had a 2.4-fold higher risk of anemia (OR 2.40, 95%CI 1.19-4.82), and those with the CT genotype had a 2.0-fold higher risk of anemia (OR 2.06, 95%CI 1.04-4.11). The association remained significant when fasting insulin, eGFR, smoking, diabetes, ACE inhibitors, sartans or diuretics treatments, use of metformin and pioglitazone were added to the model, but its independence was not retained after inclusion of fibrinogen and ESR values into the model. In conclusion, rs35767 TT allele carriers exhibited significantly higher concentrations of Hb, and lower risk of anemia compared with C allele carriers supporting the idea that IGF-1 plays a role in erythropoiesis homeostasis.

## INTRODUCTION

Anemia is a recognized risk factor for cardiovascular morbidity and mortality. There is compelling evidence that anemia is a risk factor for adverse outcomes in a number of clinical conditions including aging, diabetes mellitus, acute coronary syndromes, heart failure, chronic kidney disease [1–11]. Anemia is a multifactorial condition and several determinants may contribute to its pathogenesis, including failure of appropriate erythropoietin (EPO) production or EPO resistance, damage to EPO-producing peri-tubular cells, decreased proliferative capacity of bone marrow stem cells, impaired renal function, myelodysplasia, testosterone deficiency, chronic inflammation, iron deficiency, use of angiotensin-converting enzyme (ACE) inhibitors or angiotensin II receptor blockers (ARBs) affecting EPO production, use of metformin, causing malabsorption of vitamin B12, or pioglitazone.

In the last years, several *in vitro* and *in vivo* evidences have suggested that insulin-like growth factor-1 (IGF-1) may play an important role in erythropoiesis. Indeed, it has been demonstrated that IGF-1 promotes erythropoiesis *in vitro* stimulating the proliferation and differentiation of the late stage of primitive erythroid progenitor cells and/or early erythroid progenitor cells [12–16]. Observational studies carried out in pre-pubertal or early pubertal boys [17], nondiabetic adults [18], older (aged >65years) [19], and elderly individuals (aged >70 years) [20] have consistently shown that IGF-1 plasma levels associated with hemoglobin (Hb) concentration and anemia. Additionally, intervention studies have demonstrated that short-term treatment with recombinant human GH increased Hb in malnourished dialysis patients [21] and elderly patients [22]. Although these results suggest that IGF-1 may play an important role in regulating Hb levels under both physiological and pathological conditions, these associations may be non-causal. Factors that affect IGF-1 levels such as obesity, diabetes mellitus, adverse socioeconomic circumstances, and various disease states may themselves influence Hb concentration. The conventional approach to resolve this issue is to statistically adjust for such confounding factors, but this approach may produce misleading results, given measurement error in the assessment of confounders or the presence of unmeasured covariates, both of which lead to inadequate statistical control and residual confounding. Further, because all the studies that have assessed the association between IGF-1 levels and Hb concentration in adult subjects have a cross-sectional design [18–20], reverse causality cannot be excluded. Thus, the association between IGF-1 genotype and anemia can give an unconfounded test of whether IGF-1 levels causally influence outcomes. In an attempt to clarify this issue, we took advantage of the opportunity to study the association of an IGF-1-raising polymorphism near *IGF1* (rs35767) [23–28]. Indeed, it has been repeatedly reported that carriers of the rs35767 T allele exhibit higher levels of circulating IGF-1 [23–28]. In this study, we evaluated the association of the functional polymorphism rs35767 near *IGF1* with Hb concentration and anemia in a large cohort of well-characterized adult White Europeans.

## RESULTS

Clinical characteristics of the study group according to rs35767 genotype are presented in Table 1. The association between the rs35767 polymorphism and clinical characteristics was analysed according to both additive and recessive genetic models (Table 1). The rs35767 polymorphism did not show any significant association with age, gender, body mass index (BMI), waist circumference, smoking status (yes/no), blood pressure, fasting plasma glucose, glycated Hb (HbA1c), diagnosis of type 2 diabetes, homeostasis model assessment index of insulin resistance (HOMA-IR) index, high-sensitivity C-reactive protein (hsCRP), estimated glomerular filtration rate (eGFR), and lipids levels (Table 1). Fasting insulin levels were significantly lower in carriers of the TT genotype as compared with subjects carrying the C allele, consistent with the data of a large scale meta-analysis of genome-wide association studies of fasting insulin traits in nondiabetic individuals conducted by the Meta-Analyses of Glucose and Insulin-related traits Consortium (MAGIC) [29]. Carriers of the TT genotype exhibited significantly lower levels of ESR and fibrinogen as compared with subjects carrying the C allele (Table 1). No differences between genotypes were observed with respect to anti-hypertensive treatments, and use of metformin or pioglitazone in diabetic patients. The association of the rs35767 polymorphism with haematological parameters is reported in Table 2. Hemoglobin (Hb) concentration was significantly higher in carriers of the TT genotype as compared with C allele carriers, after adjusting for age and gender, and a lower proportion of TT carriers had anemia. No differences between genotypes were observed in hematocrit, mean corpuscular volume (MCV), mean corpuscular hemoglobin (MCH), and serum iron values. To estimate the independent contribution of rs35767 polymorphism to normochromic and normocytic anemia, we carried out a logistic regression analysis in a model which also included confounder factors affecting Hb concentration such as gender, and age. As compared with carriers of the TT genotype, those carrying the CC genotype had a 2.4-fold higher risk of having anemia (OR 2.40, 95%CI 1.19-4.82), while those carrying the CT genotype had a 2.0-fold higher risk (OR 2.06, 95%CI 1.04-4.11). The association remained significant when fasting insulin levels were added to the model (Table 3), or when eGFR, smoking status, diagnosis of diabetes, treatment with ACE inhibitors, ARBs or diuretics, use of metformin and pioglitazone in diabetic patients were added to the model (Table 3), although its independence was not retained with the further inclusion into the model of fibrinogen and erythrocytes sedimentation rate (ESR) values (Table 3).

**Table 1:** Clinical characteristics of 3286 study subjects according to the SNP rs35767 near *IGF1*

Variables	CC	CT	TT	*P*	*P* (T/T vs. C/C + C/T)
Male/Female	1088/1026	513/510	78/71	0.74	0.75
Age (yrs.)	52±13	52±14	54±10	0.22	0.22
BMI (kg/m^2^)	30.0±6.1	29.9±6.2	29.9±5.3	0.86	0.82
Waist circumference (cm)	101±14	101±15	102±14	0.77	0.17
Current smokers (%)	20.3	20.3	15.3	0.20	0.07
Systolic blood pressure (mmHg)	134±18	134±18	134±18	0.94	0.74
Diastolic blood pressure (mmHg)	81±11	81±11	81±10	0.85	0.80
Total cholesterol (mg/dl)	200±41	195±42	202±35	0.004	0.27
HDL (mg/dl)	50±14	50±14	50±14	0.76	0.90
Triglycerides (mg/dl)	136±78	134±94	137±83	0.39	0.68
HbA1c (%) [mmol/mol]	6.1±1.3 [43]	6.1±1.4 [43]	6.2±1.3 [44]	0.76	0.55
Fasting glucose (mg/dl)	100±45	110±44	111±38	0.95	0.77
Fasting insulin (μU/ml)	14±10	14±9	12±6	0.16	0.05
HOMA-IR	3.9±4.1	3.8±3.5	3.4±2.3	0.36	0.15
hsCRP (mg/l)	2.9±1.8	2.8±2.2	2.7±1.9	0.24	0.30
Fibrinogen (mg/dl)	326±83	328±87	310±72	0.07	0.02
Erythrocyte sedimentation rate (mm/h)	11±9	11±9	8±6	0.08	0.02
eGFR (ml/min/1.73m^2^)	117±38	118±40	115±32	0.55	0.38
ACE inhibitor therapy, No.(%)	559 (32.4)	278 (33.5)	49 (38.9)	0.31	0.15
Angiotensin receptor blocker therapy, No.(%)	389 (18.4)	193 (18.9)	23 (15.4)	0.60	0.36
Diuretics, No.(%)	500 (23.7)	238 (23.3)	33 (22.1)	0.89	0.69
Patients with type 2 diabetes, No.(%)	547 (25.9)	277 (27.1)	46 (30.9)	0.35	0.21
Patients with type 2 diabetes treated with metformin and/or pioglitazone, No.(%)	186 (8.8)	110 (10.7)	14 (9.3)	0.21	0.88

Data are as means ± SD. Triglycerides, hsCRP, ESR, fasting insulin concentrations, and HOMA-IR were log transformed for statistical analysis, but values in the table represent a back transformation to the original scale. Categorical variables were compared by χ^2^ test. Unpaired Student’s t test or ANOVA were used to compare differences of continuous variables between the genotypes, as appropriate. BMI: body mass index; HDL: high density lipoprotein; eGFR: estimated glomerular filtration rate; hsCRP: high sensitivity C reactive protein; HOMA-IR: homeostasis model assessment index of insulin resistance.

**Table 2:** Haematological characteristics of 3286 study subjects according to the SNP rs35767 near *IGF1*

Variables	CC	CT	TT	*P*	*P* (T/T vs. C/C + C/T)
Hemoglobin (g/dl)	13.9±1.5	13.8±4.5	14.2±1.3	0.02	0.03
Hematocrit (%)	41±4.5	41±4.5	42±4.0	0.24	0.30
Mean corpuscular volume (fl)	85±9	85±8	85±5	0.81	0.88
Mean corpuscular hemoglobin (pg)	28.5±3.2	29.9±6.2	29.9±5.3	0.28	0.24
Serum Iron (μg/dl)	84±34	80±28	86±29	0.32	0.66
Subjects with anemia, No. (%)	244 (62.9%)	135 (35.0%)	9 (2.3%)	0.03	0.02

Data are means ± SD. Categorical variables were compared by χ^2^ test. Comparisons between the groups were performed using a general linear model. *P* values refer to results after analyses with adjustment for age, and gender.

**Table 3:** Odds ratios (95% CI) by multiple logistic regression models for anemia in relation to the SNP rs35767 near IGF1

		Anemia	
Study groups	OR	95%CI	*P*
Model 1			
TT genotype (reference category)	1	---	---
CC genotype	2.40	1.19–4.82	0.01
CT genotype	2.06	1.04–4.11	0.03
Model 2			
TT genotype (reference category)	1	---	---
CC genotype	2.86	1.22–6.68	0.01
CT genotype	2.33	1.02–5.39	0.04
Model 3			
TT genotype (reference category)	1	---	---
CC genotype	2.91	1.24–6.83	0.01
CT genotype	2.40	1.03–5.55	0.04
Model 4			
TT genotype (reference category)	1	---	---
CC genotype	3.21	0.98–10.60	0.055
CT genotype	2.48	0.77–8.11	0.13

Model 1: adjusted for age, and gender. Model 2: Model 1 + fasting insulin levels. Model 3: Model 2 + eGFR, smoking status, diagnosis of diabetes, treatment with ACE inhibitors, ARBs or diuretics, use of metformin and pioglitazone in diabetic patients. Model 4: Model 3 + fibrinogen and ESR.

## DISCUSSION

There is evidence from cell biology [12–16] and observational studies [17–20] suggesting that IGF-1 may have an important role in erythropoiesis [1–6]. Previous studies have repeatedly reported that the rs35767 polymorphism near the *IGF1* gene is associated with serum IGF-1 levels [23–28].

These observations coupled with the accessibility of a carefully characterized cohort of individuals of European ancestry have provided the rationale for addressing the question of whether the rs35767 polymorphism near *IGF1* could be associated with haemoglobin concentration and anemia. We found that, individuals carrying the TT genotype, which is associated with higher circulating IGF-1 levels [23–28], exhibited significantly higher levels of haemoglobin concentration, and lower risk of having anemia as compared with carriers of the CT or CC genotype. The association between the rs35767 polymorphism near IGF1 and anemia was not affected by other confounding factors such as gender, age, smoking status, glomerular filtration rate, diagnosis of type 2 diabetes, treatment with ACE inhibitors, ARBs or diuretics, use of metformin and pioglitazone in diabetic patients. The present data, coupled with the findings of previous observational studies showing an association between low IGF-1 concentration and anemia, support the idea that IGF-1 could be an important regulator of hemoglobin concentration in adults. In spite of the elevated interindividual variability of circulating IGF-1 levels in adults, twin studies have indicated that 38% of the variance in IGF-1 concentration is attributable to genetic effects [30], suggesting the presence of a strong genetic determinant. In the Caucasian population, IGF-1 promoters containing rs35767 C allele have been shown to possess a significantly higher transcriptional activity than T allele carriers, in both in vitro and case-control studies [27, 28, 31].

In humans a role for IGF-1 similar to that of hematopoietic cytokines has been proposed (extensively discussed in Maggio M, et al. 2015 [32]); observational studies have identified IGF-1 as cause of a wide range of anemic disorders; experimental and animal studies, have demonstrated the positive influence of IGF-1 on erythropoiesis and its involvement in various steps of the hematopoietic process. Mechanistically, a decrease in IGF-1 levels may affect hemoglobin concentration by reducing erythropoiesis as a consequence of diminished IGF-1-stimulated erythroid cell growth and differentiation from bone marrow or peripheral blood [13–16]. Alternatively, because the rs35767 polymorphism near *IGF1* has been shown to influence fasting insulin in genome-wide association studies [29], and insulin stimulates the proliferation of erythroid progenitor cells and mature erythroid progenitor cells through its own receptors [16], and, possibly, via insulin/IGF-1 hybrid receptors [16–29, 33–37], it is possible to speculate that the effects of rs35767 polymorphism are mediated by insulin. Nevertheless, our findings that the association between rs35767 and anemia remained significant when fasting insulin levels were added to the model argue against this possibility. Instead, it is plausible that because IGF-1 decreases the expression of inflammatory molecules and has anti-inflammatory effects [36], a decrease in IGF-1 levels may result in higher concentrations of inflammatory proteins, which affect erythropoietin secretion, and red cell precursors survival [24]. Interestingly, we found that fibrinogen and ESR values were significantly lower in carriers of the TT genotype as compared with subjects carrying the C allele, and, more importantly, the inclusion of fibrinogen and ESR values in the logistic regression model containing all potential confounder factors, attenuated the risk for anemia of carriers of the TT genotype. Thus, an increase of anti-inflammatory action of IGF-1 may account for the lower risk of anemia observed in subjects carrying the TT genotype.

The present study has several strengths, including the large sample size encompassing men and women, the demographically homogeneous group of Italians of European ancestry, the detailed clinical characterization that allowed us to adjust for multiple confounders, the exclusion of confounding chronic conditions potentially affecting both hemoglobin concentration and inflammatory biomarkers, the clinical and laboratory variables collected by a trained staff in a standardized manner, and the centralization of laboratory analyses.

Nevertheless, some limitations should be considered in the interpretation of the current results. First, we employed the definition of anemia according to the WHO criteria [34] although other cutoffs for Hb concentration to define anemia have been proposed [38–40]. We opted for this definition since it has been widely used in previous studies thus making our results comparable to those previously reported [17–20]. A second limitation is that all variables, including circulating Hb concentration, were measured once. Although such an approach is common in clinical research, intra-individual variation in laboratory variables cannot be taken into account. Furthermore measurements of circulating IGF-1 levels were only available for a subgroup of subjects of the study cohort, thus we could not include them in our analyses. Another limitation of the present study is the cross-sectional design, making causal interpretations of associations between the rs35767 polymorphism near *IGF1* and anemia risk difficult. In addition, to avoid an unpredictable modification of the characteristics of the original sample, we chose not to exclude the individuals treated with anti-hypertensive or glucose-lowering therapies, which may influence Hb concentration. However, correction of our analyses for medication intake did not affect the results. Although the present findings are clinically and biologically plausible, a further limitation of this study is represented by the robustness of our *P* values, which do not reach a genome-wide level of significance and are, therefore, still compatible with a false-positive result. Unfortunately the rs35767 polymorphism was not among the SNPs examined by the recently published GWAS on hemoglobin or hemoglobin-related traits, and no other SNPs among those tested, can function as a good proxy for it, thus data from genome-wide association studies could not be employed to replicate the associations reported. Additionally, the present results are based on adult individuals of European ancestry, and should not be extrapolated to other ethnic groups due to differences in socio-demographic, lifestyle, anthropometric, and genetic characteristics. Furthermore, our study cohort is recruited at a referral university hospital, representing subjects carrying at least one cardio-metabolic risk factor, and, therefore, the current findings may not be extendible to the healthy general population. Overall, the present results should be considered hypothesis generating and requiring confirmation by further prospective studies on well-characterized individuals of different ethnic groups. Nonetheless, we consider our results important in attempting to understand the pathophysiological interaction between the rs35767 polymorphism near *IGF1* and Hb concentration.

## MATERIALS AND METHODS

### Study subjects

The study group comprised 3286 individuals consecutively recruited at the Department of Systems Medicine of the University of Rome-Tor Vergata and at the Department of Medical and Surgical Sciences of the University “Magna Graecia” of Catanzaro [19, 28]. The inclusion criteria were: the presence of one or more cardio-metabolic risk factors including family history of diabetes, dysglycemia/diabetes mellitus, hypertension, dyslipidemia, and overweight/obesity. Exclusion criteria comprised: history of type 1 diabetes mellitus, history of any malignant disease, end stage renal disease, heart failure, gastrointestinal affections associated with bleeding or malabsorption, macrocytic or microcytic anemia, hemoglobinopathies, hemolytic disease, autoimmune diseases, acute or chronic infections, acute or chronic pancreatitis, accumulation diseases such as amyloidosis and hemochromatosis, history of drug abuse, self-reporting alcohol consumption of >20 g/day, positivity for antibodies to hepatitis C virus (HCV) or hepatitis B surface antigen (HBsAg), and corticosteroids use.

After a 12-h fasting, all individuals underwent anthropometrical evaluation including measurements of BMI, waist circumference, readings of clinic blood pressure; a venous blood sample was drawn for laboratory determinations. The study was approved by Institutional Ethics Committees of the University of Catanzaro and Rome Tor Vergata, and written informed consent was obtained from each participant before commencing the studies in accordance with the principles of the Declaration of Helsinki.

### Analytical determinations

Haemoglobin, mean corpuscular haemoglobin (MCH), and mean corpuscular volume (MCV) were determined using an automated particle counter (Siemens Healthcare Diagnostics ADVIA^®^ 120/2120 Haematology System, Milan, Italy). Glucose, triglycerides, total and high density lipoprotein (HDL) cholesterol concentrations were determined by enzymatic methods (Roche, Basel, Switzerland). HbA1c was measured with high performance liquid chromatography using a National Glycohemoglobin Standardization Program (NGSP) certified automated analyzer (Adams HA-8160 HbA1C analyzer, Menarini, Italy). High sensitivity C reactive protein (hsCRP) levels were assessed by an automated instrument (CardioPhase^®^ hsCRP, Milan, Italy). Serum creatinine was measured by a clinical chemistry analyzer (Roche/Hitachi Modular Analytics System, P Module) using the Roche Creatinine Plus assay (Hoffman-La Roche, Basel, Switzerland). Erythrocyte sedimentation rate (ESR) was measured automatically by the stopped-flow technique in a capillary microphotometer (Alifax Test 1 System Polverara, Italy). Plasma insulin concentration was measured with a chemiluminescence-based assay (Immulite^®^, Siemens Healthcare GmbH, Erlangen, Germany).

### DNA analysis

DNA was isolated from whole blood using a commercial DNA isolation kit (Promega, Madison, WI). Screening of rs35767 polymorphism was performed using a TaqMan allelic discrimination assay (Applied Biosystems, Foster City, CA). TaqMan genotyping reaction was amplified on the iCycler Thermal Cycler and fluorescence was detected using the embedded iQ5 Multicolor Real-Time PCR Detection System (Bio-Rad Laboratories, Inc., Hercules, CA). Genotyping quality was tested by including 3 HapMap samples in each 96-well plate. The agreement rate with the HapMap database genotypes was >99%.

### Calculations

Anemia was defined according to the World Health Organization (WHO) criteria as hemoglobin <12 g/dl in women and <13 g/dl in men [34]. The homeostasis model assessment index of insulin resistance (HOMA-IR) was calculated as fasting insulin × fasting glucose/22.5 [33]. Estimated glomerular filtration rate (eGFR) was calculated by using the CKD-EPI equation [35]: eGFR = 141 x min(Scr/k, 1)^α^ x max(Scr/k, 1)^−1.209^ x 0.993^Age^ x 1.018 [if female], where Scr is serum creatinine, k is 0.7 for females and 0.9 for males, α is -0.329 for females and -0.411 for males, min indicates the minimum of Scr/k or 1, and max indicates the maximum of Scr/k or 1).

### Statistical analysis

Due to a skewed distribution, triglycerides, high sensitivity C-reactive protein (hsCRP), ESR, fasting insulin concentrations, and HOMA-IR were natural log transformed for statistical analyses. The results for continuous variables are given as means ± SD. Categorical variables were compared by χ2-test. Unpaired Student’s t test or ANOVA were used to compare differences of continuous variables between the genotypes, as appropriate. Differences of haematological variables between groups were tested after adjusting for confounding factors such as age, and gender by ANCOVA (general linear model). The Hardy-Weinberg equilibrium between the genotypes was evaluated by χ^2^ test. Genotype distributions were in Hardy-Weinberg equilibrium (*P*>0.05). The study had 80% power (for α=0.05) to detect a 2-fold or greater OR for anemia according to a recessive model. A logistic regression analysis was used to model the effect of rs35767 polymorphism on anemia and results were reported as odds ratios (ORs) with 95% CIs. Two-sided *P* value <0.05 was considered statistically significant given the high prior probabilities for association of the rs35767 polymorphism with the examined traits (the locus has already been repeatedly associated with IGF-1 levels [23–28]). All analyses were performed using SPSS (Chicago, IL, USA) software programme Version 22.0 for Windows.

## Abbreviations

EPO: erythropoietin
ACE: angiotensin-converting enzyme
ARBs: angiotensin II receptor blockers
IGF-1 or *IGF1*: insulin-like growth factor-1
Hb: hemoglobin
BMI: body mass index
HbA1c: glycated hemoglobin
HOMA-IR: homeostasis model assessment index of insulin resistance
hsCRP: high-sensitivity C-reactive protein
eGFR: estimated glomerular filtration rate
MCV: mean corpuscular volume
MCH: mean corpuscular hemoglobin
ESR: erythrocytes sedimentation rate
WHO: world health organization
SNP: single nucleotide polymorphism
